# **β**-Thalassemia Mutations among Transfusion-Dependent Thalassemia Major Patients in Northern Iraq

**DOI:** 10.4061/2010/479282

**Published:** 2010-07-05

**Authors:** Nasir A. S. Al-Allawi, Kawa M. A. Hassan, Anwar K. Sheikha, Farida F. Nerweiy, Raji S. Dawood, Jaladet Jubrael

**Affiliations:** ^1^Department of Pathology, College of Medicine and Scientific Research Center, University of Dohuk, Dohuk, Iraq; ^2^Department of Medicine, College of Medicine, Hawler Medical University, Erbil, Iraq; ^3^Scientific Research Center, University of Dohuk, Dohuk, Iraq; ^4^Thalassemia Care Center, Dohuk, Iraq

## Abstract

Molecular defects responsible for *β*-thalassemias (thal) were investigated among 254 chromosomes from 127 transfusion-dependent unrelated thalassemic patients from two provinces in Northern Iraq. Among fourteen identified mutations, the seven most common found in 88.2% of the thal chromosomes were: IVS-II-1 (G → A), IVS-I-1 (G → A), codon 8 (−AA), codon 39 (G → T), codon 8/9 (+G), codon 44 (−C), and codon 5 (−CT). There were some notable differences in frequencies of various mutations in comparison to other Eastern Mediterranean populations, as well as between the two provinces studied. The latter illustrates the relative heterogeneity of the mutations distribution in Iraq, and the need to screen other areas of the country, to ensure establishing an effective prenatal program.

## 1. Introduction

**Figure 1:**
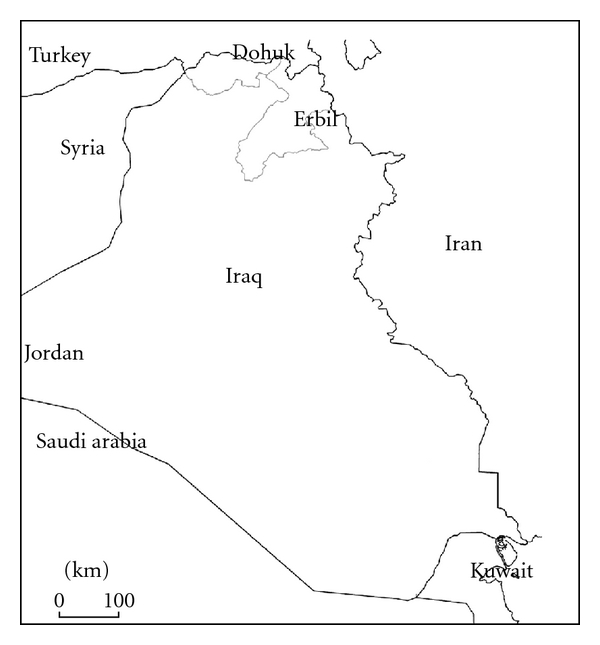
Map of Iraq, showing Erbil and Dohuk provinces (Shaded).

*β*-thalassemias (thal) are inherited defects in the rate of synthesis of *β* globin chains of hemoglobin, that are widely distributed throughout the world, with considerable frequencies in the Eastern Mediterranean countries, including Iraq [1–3]. The two most northern provinces of Iraq are Dohuk and Erbil which cover together an area of around 20 000 square kilometers (Figure 1), bordering Iran and Turkey, with a population of around 2.2 million of mostly ethnic Kurds. Thalassemia major is an important problem in these two provinces as well as other parts of the country, with more than 700 registered transfusion-dependent patients in the only two thalassemia centers in the two provinces. The huge burden imposed on the health authorities and on the patients and their families mandates that a proper preventive program is established in this region. The latter requires establishing the molecular basis of this disease in the area. An earlier smaller paper has looked at the molecular basis of this disease among carriers in one province [4], while the current study was designed to look at its basis among transfusion dependent thalassemics, with the aim of establishing an integrated prenatal program covering these two provinces.

## 2. Materials and Methods

A total of 127 unrelated transfusion dependent *β*-thal patients, registered at the Erbil (74 cases) and Dohuk (53 cases) thal care centers, northern Iraq, were recruited. The patients were all ethnic Kurds, with ages ranging between 1 and 38 years (median age of 9 years). They included 71 males and 56 females. Informed consent was obtained from all subjects, and the study was approved by the research councils of colleges of Medicine, Hawler Medical University, and Dohuk University, Iraq. A 5 mL sample was aspirated by venipuncture from each subject, anticoagulated in EDTA, frozen and then at the appropriate time had its DNA extracted by a chloroform-phenol-based method. The DNA was amplified in a multiplex reaction mixture, followed by hybridization to specific wild and mutant oligoprobes designed to detect 20 *β*-thal mutations which are encountered in Mediterranean countries (Vienna Labordiagnostica GmbH, Vienna, Austria). The *β*-thal mutations screened for, included: −87 (C→G); −30 (T→A); codon 5 (−CT); codon 6 (−A); codon 8 (−AA); codon 8/9 (+G); codon 22 (−7 bp); codon 30 (G→C); IVS-I-1 (G→A); IVS-I-2 (T→A); IVS-I-5 (G→C); IVS-I-6 (T→C); IVS-I-110 (G→A); IVS-I-116 (T→G); IVS-I (−25); codon 36/37(−T); codon 39 (C→T); codon 44 (−C); IVS-II-1 (G→A); and IVS-II-745 (C→G). The amplification, hybridization, and detection procedures were performed as recommended by the manufacturer.

## 3. Results

Among 254 *β*-thal chromosomes investigated, fourteen different mutations were identified. The seven most frequent ones constituted 88.2% of all thal defects. These mutations in order of frequency were IVS-II-1 (73 chromosomes: 28.7%), IVS-I-1 (45 chromosomes: 17.7%), codon 8 (−AA) (23 chromosomes: 9.1%), codon 8/9 (23 chromosomes: 9.1%), codon 39 (23 chromosomes: 9.1%), codon 44 (21 chromosomes, 8.3%), and codon 5 (16 chromosomes: 6.3%). Another seven mutations were less frequent or sporadic including IVS-I-6, IVS-I-5, IVS-I-110, codon 36/37, codon 30, codon 22, and IVS-II-745 (Table 1). None of the studied chromosomes had the −30 (T→A), −87 (C→G), IVS-I-2 (T→A), IVS-I-25 (25 bp del), IVS-I-116 (T→G), or codon 6 (−A) mutations. *β*-thal defects remained uncharacterized in 10 chromosome (3.9%).

 Among the 127 patients, 38 different genotypes were identified (Table 2). There were 69 homozygous patients, while the rest were compound heterozygous. Forty three of the 69 homozygous patients were the products of consanguineous marriages. The most frequent mutation among homozygous patients was IVS-II-1 followed by IVS-I-1. The most frequent compound heterozygous state was IVS-II-1/IVS-I-1 followed by IVS-I-1/codon 8/9. 

 It was observed that while IVS-II-1 is the predominant mutation in both provinces, there were notable differences in distribution of other common mutants. Erbil province contributed the larger proportion of IVS-I-1 (34/45), codon 8 (19/23) and codon 8/9 (15/23), while Dohuk contributed most cases of codon 44 (19/21) and codon 39 (16/23) (Table 1).

## 4. Discussion

This study included transfusion dependent thal major patients from two provincial thal centers covering a population of more than two millions in northern Iraq. The latter population includes mainly Ethnic Kurds, whose history suggests that they have migrated to this region through Iran more than 2500 years ago [5]. Thus, it would not be unexpected that the IVS-II-1 mutation is the most frequent mutation identified in our patients, since almost all Iranian studies report the latter mutation as the most frequent one, particularly those performed in Northwestern Iran and among Iranian Kurds [6–8]. Such high rates of IVSII-1 are not shared by Kurds of Diyarbakir in Southeastern Turkey [9] or any other of Iraq neighboring states, except for eastern Saudi Arabia and Kuwait [10, 11], which supports the notion that latter rates may also be attributable to gene flow from neighboring Iran. The higher frequency of IVS-II-1 in Iran, compared to its neighbors has led some investigators to speculate that this mutation may have originated or have undergone more efficient selection there [6]. The other most common mutations identified in the current study, were IVS-I-1 and codon 8, both Mediterranean *β*° mutations. The frequencies for both latter mutations were among the highest reported in the eastern Mediterranean region [4, 6–15]. Moreover, the majority of chromosomes carrying the latter two mutations were from Erbil, indicating further regional variations even within the same ethnic group (Iraqi Kurds). 

 Codon 8/9 (+G) is an Asian Indian *β*° mutation, which is common in the Indian subcontinent as well as Northwestern Iran and in Iranian Kurds (14.5% and 15.7%, resp.) [7, 8]. This mutation is among the common mutation in the current study and more so in Erbil nearer to Iran. Its frequency apparently decreases as we move west and north to Dohuk province and East Anatolia region of Turkey (5.1%) [12]. The mutation is less common, sporadic, or absent in other surrounding eastern Mediterranean countries [10–15]. Codon 8/9 presence in our region maybe related to trade along the ancient Silk road which passed through this part of northern Iraq. 

 Codon 39 is a Mediterranean *β*° mutation which is common mainly in the Western Mediterranean, and its frequency decreases as we move to the east except for high frequencies in Saudi Arabia and Bahrain [15], and its high frequency among our patients particularly those from Dohuk is not quite expected, as it is not shared by the Kurds of Southeastern Turkey or Western Iran at 2.8% and 1.7%, respectively [8, 9]. Codon 5, on the other hand, which has been considered a Kurdish/Iranian *β*° mutation was reported in significant proportion of cases in an earlier study from Dohuk [4], as well as Iranian Kurds [8], but not from east Anatolia region of Turkey [12]. 

 It is interesting to note that codon 44, which has been labeled as a Kurdish mutation [16], was only sporadically reported in Erbil, and the majority of cases came from Dohuk province. This is consistent with a study on Jewish Kurds originating from Dohuk region and with an earlier study on thal carriers from Dohuk which reported it as the second most frequent mutation [4, 16]. This further emphasizes the regional variation in mutation distribution among Kurds and supports the notion that the latter mutation maybe of recent origin, originating in the Dohuk region, probably in the last 2000+ years, following the Kurdish settlement in the region. Another Kurdish mutation, codon 36/37 reported among the Lurs of western Iran as well as Jewish and Iranian Kurds [8, 16, 17], was found in homozygous state in only one patient from Erbil in the current study, but not from Dohuk. Similarly, this mutation was only sporadic in East Anatolia region of Turkey bordering the two Iraqi provinces [12]. 

 Another important observation is that in contrast to many other Eastern Mediterranean populations, where the IVS-I-110 is the predominant mutation [9, 12–15], it was only seen in 2.1% of our cases, a figure which is much lower than the 27.8%, 18% and 6% reported, respectively, from southeastern Turkey, northwestern and Kurdish Iranians [7–9]. This mutation has its highest frequencies in Cyprus, but its frequency gradually decreases in countries further to the east, with a distribution pattern almost opposite to that of IVS-II-1 [10, 11, 14, 15, 18].

 It maybe argued that the high consanguinity rate among homozygous individuals may have affected the actual frequencies of some of the mutations in the current study, however, even if this is taken into account, the most frequent seven mutations will remain so, and will not thus affect the overall prenatal diagnostic program in the region.

 This study on Iraqi Kurds from northern Iraq, revealed some similarities to studies from Iranian Kurds living across the border in Iran, which consistent with the common origin of these populations. However some notable difference in the frequencies of some particular mutations between these two population and within the Iraqi Kurds are likely to be due either to recent origin of such mutations or to various selective advantages (such as Malaria) which may have had different impacts on the rates observed. Such observations further stress the need to screen other areas and ethnic groups in the country, to ensure establishing an effective prenatal diagnostic program.

## Figures and Tables

**Table 1:** The distribution of various *β*-thalassemia mutations among 254 chromosomes from Northern Iraqi provinces of Dohuk and Erbil.

Mutation	Total number of chromosomes (Erbil/Dohuk)	Number of Homozygotes (Number with consanguineous parents)	Number of Heterozygotes	Overall Frequency
IVSII.1 (G > *A*)	73 (42/31)	24 (13)	25	28.7
IVSI.1 (G > *A*)	45 (34/11)	10 (6)	25	17.7
Codon 8 (−AA)	23 (19/4)	8 (5)	7	9.1
Codon 8/9 (+G)	23 (15/8)	3 (2)	17	9.1
Codon 39 (C > *T*)	23 (7/16)	8 (5)	7	9.1
Codon 44 (−C)	21 (2/19)	7 (4)	7	8.3
Codon 5 (−CT)	16 (8/8)	3 (3)	10	6.3
IVSI.6 (T > *C*)	6 (5/1)	0	6	2.4
IVSI.5 (G > *C*)	6 (1/5)	1 (1)	4	2.4
IVSI.110 (G > *A*)	3 (3/0)	1 (0)	1	1.2
Codon 36/37 (−T)	2 (2/0)	1 (1)	0	0.8
IVSII.745 (C > *G*)	1 (1/0)	0	1	0.4
Codon 22 (−7 bp)	1 (1/0)	0	1	0.4
Codon 30 (G > *C*)	1 (0/1)	0	1	0.4
Uncharacterized	10 (8/2)	3 (3)	4	3.9

Total	254 (148/106)	69 (43)	116	

**Table 2:** Beta-thalassemia genotypes among 127 transfusion-dependent thalassemic patients enrolled in the current study.

	Genotype	Number (%)
	Homozygous	

1	IVSII.1 (G > *A*)/IVSII.1 (G > *A*)	24 (18.9)
2	IVSI.1 (G > *A*)/IVSI.1 (G > *A*)	10 (7.9)
3	Codon 8 (−AA)/Codon 8 (−AA)	8 (6.3)
4	Codon 39 (C > *T*)/Codon 39 (C > *T*)	8 (6.3)
5	Codon 44 (−C)/Codon 44 (−C)	7 (5.5)
6	Codon 8/9 (+G)/Codon 8/9 (+G)	3 (2.4)
7	Codon 5 (−CT)/Codon 5 (−CT)	3 (2.4)
8	IVSI-110 (G > *A*)/IVSI.110 (G > *A*)	1 (0.8)
9	IVSI.5 (G > *C*)/IVSI.5 (G > *C*)	1 (0.8)
10	Codon 36/37 (−T)/Codon 36/37 (−T)	1 (0.8)

	Compound Heterozygous	

11	IVSII.1 (G > *A*)/IVSI.1 (G > *A*)	9 (7.1)
12	IVSI.1 (G > *A*)/Codon 8/9 (+G)	7 (5.5)
13	IVSI.1 (G > *A*)/IVSI.6 (T > *C*)	3 (2.4)
14	IVSII.1 (G > *A*)/Codon 8/9 (+G)	3 (2.4)
15	IVSII.1 (G > *A*)/Codon 8 (−AA)	3 (2.4)
16	Codon 8/9 (+G)/Codon 5 (−CT)	3 (2.4)
17	Codon 44 (−C)/codon 5 (−CT)	3 (2.4)
18	IVSI.1 (G > *A*)/Codon 8 (−AA)	2 (1.6)
19	IVSII.1 (G > *A*)/Codon 39 (C > *T*)	2 (1.6)
20	IVSII.1 (G > *A*)/Codon 5 (−CT)	2 (1.6)
21	Codon 39 (C > *T*)/codon 44 (−C)	2 (1.6)
22	IVSII.1 (G > *A*)/IVSI.6 (T > *C*)	2 (1.6)
23	IVS.I.5 (G > *C*)/codon 8/9 (+G)	2 (1.6)
24	IVSII.1 (G > *A*)/IVSI.110 (G > *A*)	1 (0.8)
25	Codon 5 (−CT)/Codon 8 (−AA)	1 (0.8)
26	IVSI.1 (G > *A*)/Codon 39 (C > *T*)	1 (0.8)
27	IVSII.1 (G > *A*)/IVSII.745 (C > *G*)	1 (0.8)
28	IVSI.1 (G > *A*)/codon 22 (−7 bp)	1 (0.8)
29	IVSII.1 (G > *A*)/codon 30 (G > *C*)	1 (0.8)
30	IVSI.5 (G > *C*)/codon 8 (−AA)	1 (0.8)
31	IVSI.5 (G > *C*)/codon 44 (−C)	1 (0.8)
32	Codon 39 (C > *T*)/codon 5 (−CT)	1 (0.8)
33	Codon 39(C > *T*)/Codon 8/9 (+G)	1 (0.8)
34	Codon 44 (−C)/codon 8/9 (+G)	1 (0.8)
35	IVSI.1/Uncharacterized	2 (1.6)
36	IVS1.6/Uncharacterized	1 (0.8)
37	IVSII.1/Uncharacterized	1 (0.8)
38	Uncharacterized/Uncharacterized	3 (2.4)

	Total	127 (100)
